# 

**Published:** 1918-05-18

**Authors:** 


### CONTENTS.

| | PAGE |
|---|---|
| **Medical Fees to Fellow Practitioners** ... ... | 131 |
| **The Treatment of Neurasthenics**... ... ... | 132 |
| **Hospital and Institutional News** ... ... ... | 133 |
| North Staffordshire Infirmary, Hartshill—Which is the Oldest Hospital?—A High-handed Proceeding—The New Buildings and the Waiting-list at Cardiff—A Financial Policy at Walsall—Colonel White's Suggestion to Sheffield—The Endowment of a Cardiff Hospital—A New Policy at Bedford—A Humiliating Position—Expectant Mothers and Air-raids—A Secretaryship in the Family—Guardians and Hospital Accommodation—Gas and Electricity Consumption—Educational Lectures to the Wounded—An American Red Cross Worker's Impressions—The May *Searchlight*—This Week's Drug Market. | |
| **King Edward's Hospital Fund for London** ... | 137 |
| Its Majority. Some Wonderful Results. | |
| **A Ministry of Health** ... ... ... ... | 138 |
| The Scheme of the British Medical Association. | |
| **The Position of Science in Education** ... ... | 139 |
| Report of the Prime Minister's Committee. | |
| **Nature Notes from Palestine** ... ... ... | 141 |
| II. Bird Migration Seen South of Gaza. | |
| **Parliament and the Profession** ... ... ... | 142 |
| **Why Working Nurses Should Articulate** ... | 143 |
| An Open Letter from "Ierne": To the Hon. Sir Arthur Stanley, G.B.E., M.P., Chairman of the College of Nursing. | |
| **The Matrons' and Sisters' Department** ... | 144 |
| Democratic and Non-democratic Elections. | |
| Round the Hospitals. | |
| The Queen Receives Overseas Matrons—America's Chief Nurses (England and France)—New Zealand's Nursing Service—Nurse Electors: A Humorous Surprise Packet—The College Register Booming—Increased Salaries at Liverpool. | |
| **New Zealand Nursing Service** ... ... ... | 147 |
| **Women's Work in the War** ... ... ... | 148 |
| A Section of the Future Imperial War Museum. | |
| **Passing Events and Topics** ... ... ... ... | 148 |
| **King Edward's Hospital Fund for London** ... | 149 |
| Annual Meeting of the Governors and General Council. | |
| **Matrons' and Sisters' Appointments** ... ... | iv |
| **Some Annual Meetings** ... ... ... ... ... | iv |
| **The Institutional Worker** ... ... (Suppl.) | 1 |
| Fireless Cookery. Nested Covers: The Hay-box Substitute. | |

